# Predictive threshold value of peak exercise systolic blood pressure for carotid atherosclerosis among the healthy middle-aged population in China

**DOI:** 10.1016/j.pmedr.2026.103523

**Published:** 2026-06-03

**Authors:** Lili Zhao, Peng Wang, Ying Che

**Affiliations:** a Institute of Large-Scale Scientific Facilities, Beihang University, Beijing 100191, China; b Health Management Center, Peking University Third Hospital, Beijing 100191, China

**Keywords:** Cardiopulmonary exercise testing, Peak systolic blood pressure, Carotid atherosclerosis, Diagnostic cut-off value, Health check-up

## Abstract

**Objective:**

To investigate the independent association between peak systolic blood pressure (PSBP) during cardiopulmonary exercise testing and carotid atherosclerosis, and to determine the optimal cut-off value.

**Methods:**

This retrospective study included 459 individuals (aged 42–59 years, including 293 males and 166 females) who underwent health check-ups at Peking University Third Hospital from May to September 2021. Carotid atherosclerosis was defined as intima-media thickness ≥ 1.0 mm or presence of plaque. Multivariate logistic regression and receiver operating characteristic curve analysis were used.

**Results:**

PSBP was independently associated with carotid atherosclerosis (odds ratio [OR] = 1.02, *P* = 0.03). The optimal cut-off value was 183.50 mmHg (sensitivity 52.00%, specificity 71.00%). Exploratory stratified analyses showed that this parameter had predictive value in the 42–47 years age group (AUC = 0.71) and in males (AUC = 0.60), but no significant predictive value in older age groups or in females.

**Conclusions:**

PSBP was independently associated with carotid atherosclerosis, with an optimal cut-off value of 183.50 mmHg. However, its diagnostic performance was limited (sensitivity 52.00%, specificity 71.00%). Nevertheless, this parameter still has certain reference value for early risk stratification and prevention in individuals aged 42–47 years and in males.

## Highlights

• Peak systolic blood pressure is independently associated with carotid atherosclerosis.
• Our cut-off of 183.50 mmHg is below traditional exercise hypertension thresholds.
• Peak systolic blood pressure aids risk stratification in aged 42–47 and in males.

## 1 Introduction

Cardiovascular diseases (CVDs) remain the leading cause of death and disability worldwide. According to the World Health Organization (WHO), they are responsible for approximately 17.9 million deaths annually, accounting for 31% of global mortality, which imposes a substantial burden and economic pressure on society (Norouzzadeh et al., 2026). Atherosclerosis is an important pathological basis for the occurrence and progression of cardiovascular diseases (Gisterå and Hansson, 2017). The carotid artery is superficially located and easily detectable by ultrasound. Furthermore, carotid intima-media thickness and carotid plaques are significantly positively correlated with the future risk of stroke, myocardial infarction, and cardiovascular events (Ling et al., 2023; Naqvi and Lee, 2014). Therefore, it is widely regarded in clinical practice and research as a reliable indicator reflecting the health status of the systemic arterial system. Cardiopulmonary exercise testing (CPET) is the gold standard for comprehensively evaluating the integrated functional status of the cardiovascular, respiratory, and autonomic nervous systems under exercise, and can detect physiological abnormalities that are difficult to identify at rest (Guazzi et al., 2012; Balady et al., 2010; Durstenfeld et al., 2022). Systolic blood pressure (SBP) is one of the routine variables measured during exercise testing and reflects cardiovascular function and overall cardiovascular health (Pesova et al., 2023). Under physiological conditions, SBP increases linearly with increasing workload during CPET (Cauwenberghs et al., 2026) and peak SBP (PSBP) refers to the SBP value at maximal exercise. Recent studies have shown that exaggerated and blunted PSBP responses are associated with adverse clinical outcomes, and an exaggerated SBP during exercise increases the risk or future hypertension and cardiovascular events (Barlow et al., 2014; Carlén et al., 2024; Zafrir et al., 2022; Miyai et al., 2000; Schultz et al., 2013). However, sufficient evidence is still lacking regarding whether a direct association exists between PSBP and early atherosclerotic vascular damage, such as carotid structural changes (intima-media thickening or plaque formation). Moreover, previous studies have often employed fixed thresholds (≥210 mmHg in males, ≥190 mmHg in females) (Gwag et al., 2024) to define abnormal PSBP during exercise, with limited consideration of the modifying effects of physiological factors such as age, sex, resting blood pressure (BP) and exercise capacity on PSBP in the analyses. Therefore, this study aims to investigate the association between PSBP recorded during CPET and carotid atherosclerosis, evaluate their independent relationship and diagnostic performance, and determine the optimal clinical cut-off value, thereby providing a reference for utilizing exercise BP indicators to screen individuals at high risk of subclinical atherosclerosis in clinical practice.

## 2 Materials and methods

### 2.1 Study design and population

A total of 459 participants who underwent health check-ups and completed CPET at the Health Management Center, Peking University Third Hospital, from May 2021 to September 2021, were retrospectively enrolled. Based on carotid ultrasound results, they were divided into a normal carotid artery group and a carotid atherosclerosis group (participants with carotid intima-media thickness ≥ 1.0 mm or presence of plaque were included in the carotid atherosclerosis group). Inclusion criteria: (1) completed CPET; (2) asymptomatic and completed routine health examination. Exclusion criteria (White and Evans, 2001): (1)Presence of absolute contraindications to exercise stress testing as outlined in the ACSM's Guidelines for Exercise Testing and Prescription (11th edition), (2) Diagnosis of coronary artery disease, heart failure, pulmonary hypertension, valvular heart disease, cardiomyopathy, severe ventricular arrhythmia, myocarditis, or pericarditis, (3) Severe respiratory diseases such as chronic obstructive pulmonary disease or severe asthma, (4) Uncontrolled hyperthyroidism or hypothyroidism, (5) Uncontrolled diabetes mellitus, (6) Severe electrolyte disturbances, (7) Severe hypertension [SBP ≥160 mmHg or diastolic blood pressure (DBP) ≥100 mmHg]**,** regardless of antihypertensive medication use, (8) Severe anemia (haemoglobin concentration < 60 g/L), (9) Abnormal liver function, renal function, or myocardial enzyme profile, (10) Major abnormalities in electrocardiogram, echocardiography, or carotid ultrasound findings, (11) Presence of infectious diseases. This study was approved by the Medical Ethics Committee of Peking University Third Hospital (Approval No. 2025-134-02). Written informed consent was obtained from all participants.

### 2.2 Measures

CPET was performed using the Quark PFT4 ergo cardiopulmonary exercise testing system (COSMED, Italy). Prior to CPET, multi-flow gas calibration was conducted, upon successful calibration, the CPET proceeded. CPET was conducted in accordance with relevant standards (Glaab and Taube, 2022): (1) Cardiopulmonary parameters were monitored and recorded throughout the test. After resting quietly for 3 min, participants performed unloaded warm-up cycling at 60 r/min for 3 min, followed by incremental load cycling until they could no longer maintain the pedaling frequency. BP was primarily measured using the automated module of the Cosmed system (with manual auscultation when necessary) and recorded at rest, during warm-up, every 3 min during exercise, at peak, and during recovery (1st, 2nd, 4th, 6th minute). PSBP was defined as the SBP value recorded at the moment of maximum exercise load. When SBP appears alone in the text without special specification, it refers to resting SBP. Oxygen uptake (VO₂): VO₂ was collected using the breath-by-breath method and automatically calculated (Cosmed Quark PFT ergo, Omnia software). The percentage of predicted peak VO₂ was calculated using the Wasserman/Hansen prediction equations. Heart rate (HR): Maximal HR (HRmax) was defined as the highest HR value occurring during CPET, continuously recorded by electrocardiogram. Predicted HRmax was calculated using the Fox formula (220 – age). The percentage of predicted HRmax was calculated as (measured HRmax / predicted HRmax) × 100%. Oxygen uptake efficiency slope (OUES) is the slope of linear regression of VO₂ against log₁₀ (minute ventilation). After termination of the exercise test, electrocardiogram (ECG) monitoring continued for at least 6 min or until pre-exercise conditions were restored, (2) Exercise test termination criteria were as follows (Gibbons et al., 1997; Gibbons et al., 2002; Albouaini et al., 2007): onset of angina pectoris, neurological symptoms such as ataxia, dizziness, or unsteady gait, signs of hypoperfusion such as cyanosis or pallor, a decrease in SBP > 10 mmHg or a rapid increase >220 mmHg with increasing workload, abnormal ECG manifestations (ST-segment elevation ≥0.1 mV, horizontal or downsloping ST-segment depression >0.2 mV, or worsening of pre-existing ST-segment depression, marked axis deviation, severe arrhythmias such as sustained ventricular tachycardia, frequent polymorphic ventricular premature beats, supraventricular tachycardia, and other arrhythmias that may compromise hemodynamic stability or be life-threatening, such as ventricular fibrillation), and voluntary request by the participant to stop.

General information included age, sex, body mass index (BMI), SBP, DBP, and HR. Body weight was measured in kilograms using an electronic scale, and standing height was measured in meters with a wall-mounted electronic stadiometer, and BMI was calculated as weight in kilograms divided by the square of height in meters (kg/m^2^). Resting BP was measured once at the end of a 3 min rest period (before exercise initiation) with the participant seated on the cycle ergometer, arm positioned at heart level, using the automated blood pressure module of the Cosmed Quark PFT ergo system. Blood biochemical parameters included fasting plasma glucose (Glu), total cholesterol (TC), triglycerides (TG), low-density lipoprotein cholesterol (LDL-C), high-density lipoprotein cholesterol (HDL-C), and creatinine.

Carotid ultrasound was performed by experienced sonographer using Hitachi HI VISION Ascendus colour Doppler ultrasound system. Measurement sites included distal common carotid artery, bifurcation, and the proximal internal carotid artery bilaterally. Carotid intima-media thickness (CIMT) was the mean of six sites (three per side). CIMT ≥1.0 mm indicated thickening. Plaque was defined as focal thickening of the intima-media layer ≥1.5 mm, or focal thickening exceeding 50% of the adjacent CIMT value, or a focal echogenic structure protruding into the lumen. Carotid ultrasound was performed within one week before CPET.

### 2.3 Statistical analysis

Normally distributed continuous data are presented as mean ± standard deviation, and comparisons between groups were performed using the independent samples *t*-test (Welch's correction was applied in case of heterogeneity of variance). Non-normally distributed continuous data are expressed as median (interquartile range), with group comparisons performed using the Mann–Whitney *U* test. Categorical data are presented as frequency (percentage), and comparisons between groups were performed using the chi-squared test. Normality of the data was tested using the Kolmogorov-Smirnov test. All tests were two-sided, and a *P*-value <0.05 was considered statistically significant. Univariate logistic regression analysis was performed to evaluate the association between PSBP and carotid atherosclerosis. Subsequently, binary multivariate logistic regression analysis was used to investigate the independent association between PSBP and carotid atherosclerosis. Variables with *p* < 0.05 in univariate analysis or those considered clinically important based on previous literature were entered into the multivariate logistic regression model. Multicollinearity among independent variables was assessed using the variance inflation factor (VIF). The receiver operating characteristic (ROC) curve was used to evaluate the predictive value of PSBP for carotid atherosclerosis, and the area under the curve and its 95% confidence interval were calculated. The optimal diagnostic cut-off value was determined by the maximum Youden index, and the corresponding sensitivity, specificity, and accuracy were calculated. The Institutional Review Board of Peking University Third Hospital approved the study. Statistical analysis was performed using SPSS version 27.0 software.

## 3 Results

### 3.1 Comparison of demographic information and biochemical parameters

The comparison of demographic information and biochemical parameters between the two groups is presented in Table 1. Compared with the normal carotid artery group, the carotid atherosclerosis group was significantly older, had a higher proportion of males, higher proportions of patients with a history of hypertension and diabetes, and had a higher BMI, with all differences being statistically significant (*P* < 0.05). The carotid atherosclerosis group had significantly higher levels of creatinine, TG, and Glu, and significantly lower HDL-C levels than the normal carotid artery group (*P* < 0.05). However, no statistically significant differences were observed in office blood pressure, TC or LDL-C levels between the two groups (*P* > 0.05).

**Table 1:** Comparison of demographic and biochemical characteristics between participants with and without carotid atherosclerosis among healthy middle-aged adults in China (2021).

Variable	Normal carotid artery group (*n* = 212)	Carotid atherosclerosis group (*n* = 247)	t/χ^2^/Z	P-value
Demographic information				
Age (years)	48.42 ± 4.40	52.28 ± 4.68	−9.06	<0.01
Sex			42.18	<0.01
- Male	102 (48.11)	191 (77.33)		
- Female	110 (51.89)	56 (22.67)		
History of hypertension, n (%)			15.40	<0.01
- Yes	15 (7.35)	49 (20.50)		
- No	189 (92.65)	190 (79.50)		
History of diabetes, n (%)			6.66	0.01
- Yes	3 (1.47)	15 (6.36)		
- No	201 (98.53)	221 (93.64)		
Office blood pressure classification, n (%)			3.53	0.06
- Normal	180 (85.71)	195 (78.95)		
- Elevated	30 (14.29)	52 (21.05)		
BMI (kg/m^2^)	24.15 ± 3.01	24.92 ± 2.91	−2.79	0.01
Biochemical parameters				
Creatinine (μmol/L)	78.42 ± 13.09	83.68 ± 12.07	−4.48	<0.01
TC (mmol/L)	5.12 ± 0.81	5.09 ± 0.98	0.46	0.65
TG (mmol/L)	1.19 (0.90, 1.72)	1.35 (0.98, 1.95)	−2.57	0.01
LDL-C (mmol/L)	3.08 ± 0.65	3.11 ± 0.80	−0.41	0.68
HDL-C (mmol/L)	1.37 ± 0.35	1.27 ± 0.30	3.49	<0.01
Glu (mmol/L)	5.00 (4.70, 5.40)	5.20 (4.90, 5.70)	−4.66	<0.01

Notes: BMI, body mass index; TC, total cholesterol; TG, triglycerides; LDL-C, low-density lipoprotein cholesterol; HDL-C, high-density lipoprotein cholesterol; Glu, fasting plasma glucose. P values were obtained from independent samples t-test (Welch's correction for unequal variances) or Mann-Whitney U test for continuous variables, and chi-squared test for categorical variables.

### 3.2 Comparison of resting and CPET parameters between the two groups

The comparison of resting and CPET parameters between the two groups is presented in Table 2. Compared with the normal carotid artery group, the carotid atherosclerosis group had significantly higher SBP, DBP, PSBP, peak diastolic blood pressure (PDBP), and OUES, peak workload, absolute peak VO₂, absolute VO₂ at anaerobic threshold, and body-weight-indexed VO₂ at anaerobic threshold, with all differences being statistically significant (P < 0.05). However, no statistically significant differences were observed between the two groups in HR, HRmax as a percentage of the predicted maximum (% predicted HRmax), maximal oxygen uptake as a percentage of predicted value (VO2 Max/Pred), oxygen uptake at anaerobic threshold as a percentage of predicted value (VO₂ AT/Pred), breathing reserve (BR), body-weight-indexed peak VO₂, or ventilatory equivalent for carbon dioxide slope (VE/VCO₂ slope) (*P* > 0.05).

**Table 2:** Comparison of resting and cardiopulmonary exercise testing parameters between participants with and without carotid atherosclerosis among healthy middle-aged adults in China (2021).

Variable	Normal carotid artery group (n = 212)	Carotid atherosclerosis group(n = 247)	t/χ^2^/Z	P-value
Resting parameters				
SBP (mmHg)	117.75 ± 14.39	123.74 ± 15.60	−4.25	<0.01
DBP (mmHg)	76.02 ± 10.32	79.27 ± 10.10	−3.40	<0.01
HR (bpm)	80.92 ± 12.27	80.70 ± 11.47	0.20	0.84
CPET parameters				
PSBP (mmHg)	172.06 ± 22.81	183.41 ± 24.91	−5.01	<0.01
PDBP (mmHg)	84.71 ± 11.74	86.79 ± 10.10	−2.02	0.04
%predicted HRmax	87.82 ± 9.05	87.36 ± 9.64	0.52	0.60
VO2 Max/Pred (%)	79.79 ± 16.28	80.63 ± 15.57	−0.56	0.58
VO2 AT/Pred (%)	50.87 ± 12.52	52.13 ± 12.91	−1.05	0.29
OUES	1742.87 ± 498.88	1913.62 ± 474.97	−3.61	<0.01
peak workload (W)	137.94 ± 42.33	148.65 ± 34.48	−2.93	<0.01
absolute peak VO₂ (L/min)	1.54 ± 0.48	1.68 ± 0.42	−3.26	<0.01
body-weight-indexed peak VO₂ (mL/kg/min)	22.84 ± 5.36	23.61 ± 5.18	−1.56	0.12
absolute VO₂ at anaerobic threshold (L/min)	0.97 ± 0.32	1.08 ± 0.32	−3.62	<0.01
body-weight-indexed VO₂ at anaerobic threshold (mL/kg/min)	14.47 ± 3.87	15.24 ± 3.94	−2.10	0.04
BR	0.52 ± 0.13	0.50 ± 0.14	1.57	0.12
VE/VCO₂ slope	25.40 ± 3.44	25.66 ± 4.30	−0.72	0.47

Notes: SBP, systolic blood pressure; DBP, diastolic blood pressure; HR, heart rate; CPET, cardiopulmonary exercise testing; PSBP, peak systolic blood pressure; PDBP, peak diastolic blood pressure; %predicted HRmax, HRmax as a percentage of the predicted maximum; VO2 Max/Pred, maximal oxygen uptake as a percentage of predicted value; VO2 AT/Pred, oxygen uptake at anaerobic threshold as a percentage of predicted value; OUES, oxygen uptake efficiency slope; VO₂, oxygen uptake; BR, breathing reserve; VE/VCO₂ slope, ventilatory equivalent for carbon dioxide slope. P values were obtained from independent samples t-test (Welch's correction for unequal variances) or Mann-Whitney U test for continuous variables.

### 3.3 Association and predictive value of PSBP for carotid atherosclerosis

Univariate logistic regression analysis showed that PSBP was significantly associated with carotid atherosclerosis (odds ratio [OR] = 1.02, *P* < 0.01) (Table 3). Binary multivariate logistic regression analysis showed that PSBP was an independent risk factor for carotid atherosclerosis (OR = 1.02, *P* = 0.03). Age was also an independent risk factor for carotid atherosclerosis (OR = 1.19, *P* < 0.01) The associations between the remaining variables and carotid atherosclerosis were not statistically significant (P > 0.05). Details of the multivariate analysis are shown in Table 4.

**Table 3:** Univariate logistic regression analysis of PSBP for carotid atherosclerosis among healthy middle-aged adults in China (2021).

Variable	B	SE	OR	P-value
PSBP (mmHg)	0.02	0.00	1.02	<0.01

Notes: PSBP, peak systolic blood pressure; OR, odds ratio; B, regression coefficient; SE, standard error. *P* values were obtained from univariate logistic regression analysis.

**Table 4:** Multivariate logistic regression analysis of factors associated with carotid atherosclerosis among healthy middle-aged adults in China (2021).

Variable	Β	SE	OR	p-value
Demographic information				
Age (years)	0.17	0.03	1.19	<0.01
Sex (male/female)	−0.48	0.39	0.62	0.22
BMI (kg/m^2^)	−0.03	0.05	0.98	0.61
Clinical parameters				
History of hypertension (yes/no)	0.60	0.38	1.82	0.12
History of diabetes (yes/no)	0.42	0.71	1.52	0.56
LDL-C (mmol/L)	0.12	0.17	1.13	0.46
TG (mmol/L)	0.11	0.12	1.12	0.36
HDL-C (mmol/L)	−0.22	0.45	0.80	0.62
Resting blood pressure				
SBP (mmHg)	0.01	0.01	1.01	0.62
DBP (mmHg)	0.00	0.02	1.00	0.88
CPET parameters				
PSBP (mmHg)	0.02	0.01	1.02	0.03
PDBP (mmHg)	−0.02	0.02	0.98	0.20
Peak workload (W)	0.00	0.01	1.00	0.68
OUES	0.00	0.00	1.00	0.69

Notes: BMI, body mass index; LDL-C, low-density lipoprotein cholesterol; TG, triglycerides; HDL-C, high-density lipoprotein cholesterol; SBP, systolic blood pressure; DBP, diastolic blood pressure; CPET, cardiopulmonary exercise testing; PSBP, peak systolic blood pressure; PDBP, peak diastolic blood pressure; OUES, oxygen uptake efficiency slope; B, regression coefficient; SE, standard error; OR, odds ratio. P values were obtained from binary multivariate logistic regression analysis. Multicollinearity diagnostics showed that cardiopulmonary function variables, including absolute peak oxygen uptake (VO₂), absolute VO₂ at anaerobic threshold, maximal oxygen uptake as a percentage of predicted value (VO₂ Max/Pred) and peak workload, exhibited high multicollinearity (variance inflation factor [VIF] > 10). To avoid model instability, these variables were not simultaneously entered into the regression equation. Only peak workload, which had stronger clinical relevance, was included in the final model. Sensitivity analysis confirmed that the core conclusions remained unchanged when other cardiopulmonary variables were substituted (Supplementary Material S1).

**Fig. 1:**
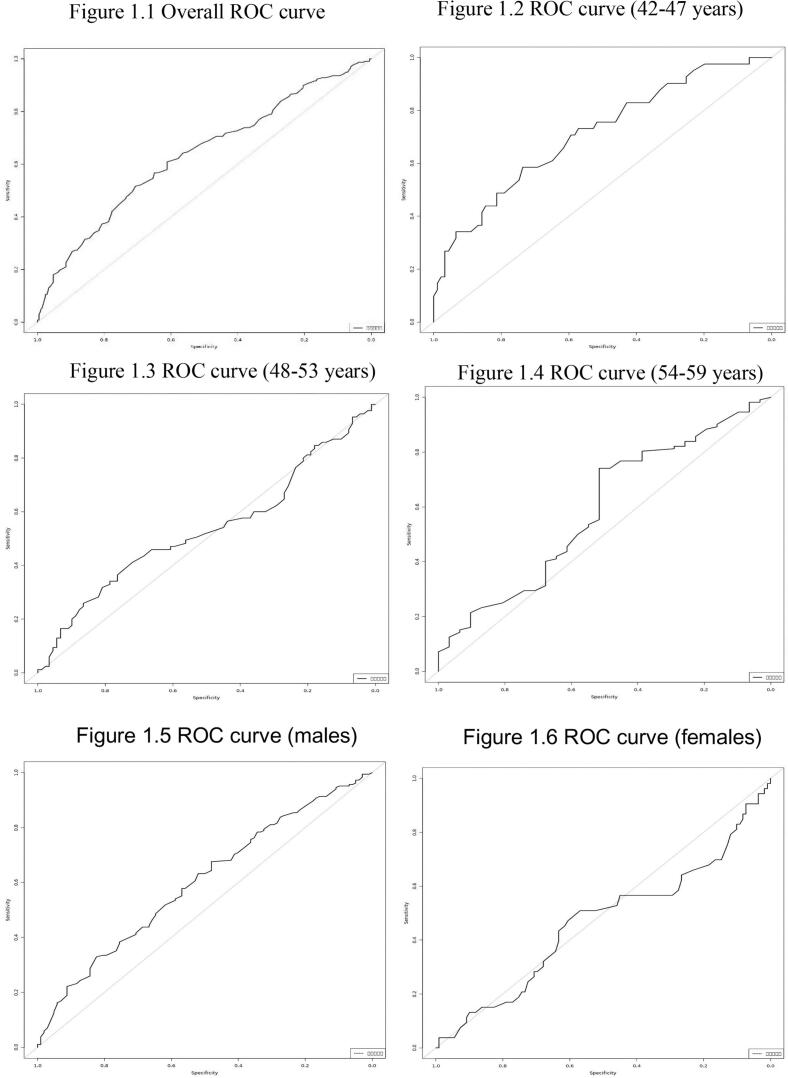
Receiver operating characteristic curves of PSBP for predicting carotid atherosclerosis among healthy middle-aged adults in China (2021). Notes: PSBP, peak systolic blood pressure; ROC curve, receiver operating characteristic curve. P values were obtained from receiver operating characteristic curve analysis.

ROC curve analysis (Fig. 1) showed that the area under the curve (AUC) of PSBP for predicting carotid atherosclerosis was 0.63 (P < 0.01). The optimal diagnostic cut-off value determined by the maximum Youden index was 183.50 mmHg, with a sensitivity of 52.00% and a specificity of 71.00% for predicting carotid atherosclerosis (Fig. 1.1). Further exploratory stratified analysis was performed by dividing participants into three groups according to equal age intervals. The results showed that in the 42–47 years age group, PSBP had significant predictive value for carotid atherosclerosis (Fig. 1.2, *n* = 134, AUC = 0.71, *P* < 0.01). In the 48–53 years age group (Fig. 1.3, *n* = 178, AUC = 0.47, *P* = 0.45) and the 54–59 years age group (Fig. 1.4, *n* = 147, AUC = 0.41, *P* = 0.13), no significant predictive value of PSBP for carotid atherosclerosis was observed. Sex-stratified analysis showed that in males, PSBP had a significant predictive value for carotid atherosclerosis (Fig. 1.5, AUC = 0.60, *P* = 0.01), in females, PSBP had no significant predictive value (Fig. 1.6, AUC = 0.47, *P* = 0.48).

## 4 Discussion

This study investigated the association between PSBP and carotid atherosclerosis. PSBP was significantly higher in the atherosclerosis group than in the normal group (183.41 ± 24.91 mmHg vs. 172.06 ± 22.81 mmHg, P < 0.01). After adjusting for age, sex, BMI, lipids, BP, medical history, and exercise capacity, each 1 mmHg increase in PSBP was associated with a 2% increase in the risk of carotid atherosclerosis. Previous studies reported similar associations (Jae et al., 2006; Yoon and Kim, 2024) but did not propose a screening cut-off. This study confirmed this association and proposed an optimal cut-off value of PSBP (183.50 mmHg) for predicting carotid atherosclerosis, along with its age- and sex-stratified characteristics. Repeated exercise-induced blood pressure elevation damages vascular endothelium, leading to dysfunction (reduced nitric oxide, dysregulation of the renin-angiotensin system, and endothelin imbalance), which in turn triggers oxidative stress, inflammatory responses, and vascular smooth muscle cell proliferation, ultimately resulting in carotid intima-media thickening or plaque formation (Çelik et al., 2025; De Bartolo et al., 2025; Xin et al., 2024). Both groups exhibited reduced exercise capacity. Reduced exercise capacity is often accompanied by sympathetic overactivity and an enhanced exercise pressor reflex, changes that tend to increase the exercise blood pressure response (Qin et al., 2022). However, there was no significant difference between the two groups in VO2 Max/Pred or weight-corrected peak VO₂, indicating comparable exercise capacity. Therefore, impaired exercise capacity alone cannot explain the difference in PSBP between the two groups. Notably, the atherosclerosis group had higher PSBP, OUES, peak workload, and absolute peak VO₂, suggesting seemingly better cardiopulmonary function. Framingham data show that higher exercise SBP can result from either favorable factors (better cardiopulmonary function) or unfavorable factors (increased arterial stiffness) (Nayor et al., 2023). This group had a higher proportion of males (77.33% vs. 48.11%) and greater body weight, consistent with the aforementioned indices. Thus, the higher OUES, peak workload, and absolute peak VO₂ in this group are likely driven by demographic and body size characteristics rather than truly superior cardiopulmonary function (Deiwert et al., 2024; Kernizan et al., 2022; Milani et al., 2023). Multivariate analysis showed that the association between OUES and atherosclerosis became non-significant after adjusting for sex, confirming that elevated PSBP primarily reflects pre-existing vascular dysfunction.

The optimal diagnostic cut-off value of PSBP for carotid atherosclerosis derived from ROC curve analysis (approximately 183.50 mmHg) was significantly lower than the currently used clinical diagnostic threshold for “exercise hypertension” (≥ 210 mmHg in males and ≥ 190 mmHg in females) (Gwag et al., 2024; Kosowski and Aleksandrowicz, 2024; Janssens et al., 2025), suggesting that, for the purpose of identifying subclinical vascular damage (such as carotid atherosclerosis) in clinical practice, a lower hemodynamic cut-off point than the traditional definition of exercise hypertension may be needed to identify individuals at high risk. PSBP may serve as an auxiliary reference indicator for identifying individuals at high risk of carotid atherosclerosis in clinical settings. Therefore, for individuals whose PSBP reaches this level after exercise, further screening for relevant vascular conditions is recommended. Exploratory analysis based on equal age intervals revealed that the predictive value of PSBP for carotid atherosclerosis was observed only in the 42–47 years age group, with no significant predictive efficacy in older populations. Wenner (Wenner et al., 2024) reported that endothelial function (FMD) in women exhibits a declining inflection point at age 47, with accelerated decline thereafter, while aortic stiffness (cfPWV) remains relatively stable before age 48 and shows an independent positive correlation with age after this threshold. Another study (Kim et al., 2024) also observed a steeper increase in arterial stiffness in women after age 50. In the present study, the 42–47 years age range, in which PSBP demonstrated predictive value for carotid atherosclerosis, coincides precisely with the period preceding the onset of vascular functional changes reported in these studies. This finding suggests that PSBP may serve as an indicator reflecting early vascular functional status in this age group, thereby exhibiting a certain predictive capacity for carotid atherosclerosis. In individuals aged 48 years and older, however, as vascular function enters a phase of accelerated change, structural vascular alterations may become prevalent, and the independent predictive efficacy of PSBP may consequently be attenuated. Sex-stratified analysis showed that the predictive value of PSBP for carotid atherosclerosis was significant in males but not in females. However, the AUC in males was only 0.60, so the results should be interpreted cautiously. The mechanisms underlying this sex difference remain unclear and warrant further investigation.

This study has several limitations. Firstly, as an observational study, it did not fully account for the potential impact of lifestyle factors such as smoking, alcohol consumption, and physical activity on carotid atherosclerosis. Future studies should further control for these confounding factors. Secondly, the study population was limited to relatively healthy middle-aged Chinese individuals, therefore, generalization of the findings to other populations should be approached with caution. In addition, as a single-center cross-sectional study, this research cannot establish causality. Further validation through multicenter prospective cohort studies or interventional trials is warranted.

## 5 Conclusion

In conclusion, this study demonstrates that PSBP during CPET is significantly associated with carotid atherosclerosis, with an optimal cut-off value of 183.50 mmHg. However, its diagnostic performance was modest. Exploratory stratified analyses suggested that PSBP had predictive value only in individuals aged 42–47 years and in males, with no significant predictive value in older age groups or in females. These findings support the integration of exercise blood pressure monitoring into early risk screening systems for subclinical atherosclerosis and provide a reference for developing prevention strategies centered on early intervention. Future research should prioritize prospective and interventional studies to validate the clinical applicability of this cut-off value.

## CRediT authorship contribution statement

**Lili Zhao:** Writing – review & editing, Writing – original draft, Visualization, Software, Methodology, Formal analysis, Data curation. **Peng Wang:** Writing – review & editing, Supervision, Project administration, Funding acquisition, Conceptualization. **Ying Che:** Writing – review & editing, Supervision, Resources, Funding acquisition, Conceptualization.

## Informed consent statement

Not applicable.

## Institutional review board statement

The study was conducted in accordance with the Declaration of Helsinki and approved by the Medical Ethics Committee of Peking University Third Hospital[Approval No: (2025) Medical Ethics Review No. (134–02).

## Funding

This study was supported by the National Science and Technology Innovation 2030, Noncommunicable Chronic Diseases-National Science and Technology Major Project (Grant No. 2024ZD0524300, 2024ZD0524301), The Cohort Construction Project of Peking University Third Hospital (BYSYDL2024010).

## Declaration of competing interest

The authors declare that they have no known competing financial interests or personal relationships that could have appeared to influence the work reported in this paper.

## Data Availability

Data will be made available on request.
