# Supplementary material for: Predictive threshold value of peak exercise systolic blood pressure for carotid atherosclerosis among the healthy middle-aged population in China
Source: Prev Med Rep. 2026 Jun 3;67:103523. doi: 10.1016/j.pmedr.2026.103523 (PMC13264132; doi:10.1016/j.pmedr.2026.103523)
Supplement: Supplementary file 1 — Supplementary material [file mmc1.docx]

Sensitivity analysis 1: Multivariate logistic regression analysis of factors associated with carotid atherosclerosis among healthy middle-aged adults in China (2021)

| Variable | Β | SE | OR | p-value |
| --- | --- | --- | --- | --- |
| Demographic information |  |  |  |  |
| Age (years) | 0.17 | 0.03 | 1.19 | <0.001 |
| Sex (male/female) | -0.45 | 0.36 | 0.64 | 0.21 |
| BMI (kg/m²) | -0.03 | 0.05 | 0.97 | 0.56 |
| Clinical parameters |  |  |  |  |
| History of hypertension (yes/no) | 0.60 | 0.38 | 1.82 | 0.12 |
| History of diabetes (yes/no) | 0.41 | 0.72 | 1.50 | 0.57 |
| LDL-C (mmol/L) | 0.13 | 0.17 | 1.14 | 0.43 |
| TG (mmol/L) | 0.12 | 0.12 | 1.13 | 0.33 |
| HDL-C (mmol/L) | -0.23 | 0.45 | 0.80 | 0.62 |
| Resting blood pressure |  |  |  |  |
| SBP (mmHg) | 0.01 | 0.01 | 1.01 | 0.56 |
| DBP (mmHg) | 0.00 | 0.02 | 1.00 | 0.87 |
| CPET parameters |  |  |  |  |
| PSBP (mmHg) | 0.02 | 0.01 | 1.02 | 0.03 |
| PDBP (mmHg) | -0.02 | 0.02 | 0.98 | 0.18 |
| absolute peak VO₂ (L/min) | 0.49 | 0.53 | 1.64 | 0.35 |
| OUES | 0.00 | 0.00 | 1.00 | 0.96 |

Notes: BMI, body mass index; LDL-C, low-density lipoprotein cholesterol; TG, triglycerides; HDL-C, high-density lipoprotein cholesterol; SBP, systolic blood pressure; DBP, diastolic blood pressure; CPET, cardiopulmonary exercise testing; PSBP, peak systolic blood pressure; PDBP, peak diastolic blood pressure; OUES, oxygen uptake efficiency slope; VO₂, Oxygen uptake; B, regression coefficient; SE, standard error; OR, odds ratio. P values were obtained from binary multivariate logistic regression analysis. Sensitivity analysis showed that substituting absolute peak VO₂ for peak workload did not alter the main findings.

Sensitivity analysis 2: Multivariate logistic regression analysis of factors associated with carotid atherosclerosis among healthy middle-aged adults in China (2021)

| Variable | Β | SE | OR | p-value |
| --- | --- | --- | --- | --- |
| Demographic information |  |  |  |  |
| Age (years) | 0.17 | 0.03 | 1.19 | <0.001 |
| Sex (male/female) | -0.62 | 0.34 | 0.54 | 0.07 |
| BMI (kg/m²) | 0.01 | 0.05 | 1.01 | 0.87 |
| Clinical parameters |  |  |  |  |
| History of hypertension (yes/no) | 0.63 | 0.39 | 1.88 | 0.10 |
| History of diabetes (yes/no) | 0.40 | 0.71 | 1.49 | 0.57 |
| LDL-C (mmol/L) | 0.12 | 0.17 | 1.13 | 0.46 |
| TG (mmol/L) | 0.12 | 0.12 | 1.13 | 0.33 |
| HDL-C (mmol/L) | -0.32 | 0.45 | 0.72 | 0.48 |
| Resting blood pressure |  |  |  |  |
| SBP (mmHg) | 0.01 | 0.01 | 1.01 | 0.65 |
| DBP (mmHg) | 0.00 | 0.02 | 1.00 | 0.90 |
| CPET parameters |  |  |  |  |
| PSBP (mmHg) | 0.02 | 0.01 | 1.02 | 0.03 |
| PDBP (mmHg) | -0.02 | 0.02 | 0.98 | 0.21 |
| body-weight-indexed VO₂ at anaerobic threshold (mL/kg/min) | 0.09 | 0.05 | 1.09 | 0.07 |
| OUES | 0.00 | 0.00 | 1.00 | 0.59 |

Notes: BMI, body mass index; LDL-C, low-density lipoprotein cholesterol; TG, triglycerides; HDL-C, high-density lipoprotein cholesterol; SBP, systolic blood pressure; DBP, diastolic blood pressure; CPET, cardiopulmonary exercise testing; PSBP, peak systolic blood pressure; PDBP, peak diastolic blood pressure; OUES, oxygen uptake efficiency slope; VO₂, Oxygen uptake; B, regression coefficient; SE, standard error; OR, odds ratio. P values were obtained from binary multivariate logistic regression analysis. Sensitivity analysis showed that substituting body-weight-indexed VO₂ at anaerobic threshold for peak workload did not alter the main findings.

Sensitivity analysis 3: Multivariate logistic regression analysis of factors associated with carotid atherosclerosis among healthy middle-aged adults in China (2021)

| Variable | Β | SE | OR | p-value |
| --- | --- | --- | --- | --- |
| Demographic information |  |  |  |  |
| Age (years) | 0.17 | 0.03 | 1.19 | <0.001 |
| Sex (male/female) | -0.52 | 0.34 | 0.60 | 0.13 |
| BMI (kg/m²) | -0.04 | 0.05 | 0.96 | 0.40 |
| Clinical parameters |  |  |  |  |
| History of hypertension (yes/no) | 0.65 | 0.39 | 1.92 | 0.09 |
| History of diabetes (yes/no) | 0.40 | 0.71 | 1.49 | 0.58 |
| LDL-C (mmol/L) | 0.13 | 0.17 | 1.14 | 0.44 |
| TG (mmol/L) | 0.12 | 0.12 | 1.13 | 0.31 |
| HDL-C (mmol/L) | -0.32 | 0.45 | 0.72 | 0.48 |
| Resting blood pressure |  |  |  |  |
| SBP (mmHg) | 0.01 | 0.01 | 1.01 | 0.57 |
| DBP (mmHg) | 0.00 | 0.02 | 1.00 | 0.83 |
| CPET parameters |  |  |  |  |
| PSBP (mmHg) | 0.02 | 0.01 | 1.02 | 0.03 |
| PDBP (mmHg) | -0.02 | 0.02 | 0.98 | 0.21 |
| absolute AT VO₂ (L/min) | 1.32 | 0.70 | 3.73 | 0.06 |
| OUES | 0.00 | 0.00 | 1.00 | 0.43 |

Notes: BMI, body mass index; LDL-C, low-density lipoprotein cholesterol; TG, triglycerides; HDL-C, high-density lipoprotein cholesterol; SBP, systolic blood pressure; DBP, diastolic blood pressure; CPET, cardiopulmonary exercise testing; PSBP, peak systolic blood pressure; PDBP, peak diastolic blood pressure; OUES, oxygen uptake efficiency slope; VO₂, Oxygen uptake; B, regression coefficient; SE, standard error; OR, odds ratio. P values were obtained from binary multivariate logistic regression analysis. Sensitivity analysis showed that substituting absolute AT VO₂ for peak workload did not alter the main findings.
